# Exploring the Continuum of Vaccine Hesitancy Between African American and White Adults: Results of a Qualitative Study

**DOI:** 10.1371/currents.outbreaks.3e4a5ea39d8620494e2a2c874a3c4201

**Published:** 2016-12-29

**Authors:** Sandra Quinn, Amelia Jamison, Donald Musa, Karen Hilyard, Vicki Freimuth

**Affiliations:** Department of Family Science, University of Maryland School of Public Health, College Park, Maryland, USA; Senior Associate Director, Maryland Center for Health Equity, University of Maryland School of Public Health, College Park, Maryland, USA; Center for Health Equity, University of Maryland School of Public Health, College Park, Maryland, USA; University Center for Social and Urban Research, University of Pittsburgh, Pittsburgh, Pennsylvania, USA; Department of Health Promotion and Behavior, University of Georgia College of Public Health, Athens, Georgia, USA; Center for Health and Risk Communication, University of Georgia, Athens, Georgia, USA

## Abstract

Vaccine delay and refusal present very real threats to public health. Since even a slight reduction in vaccination rates could produce major consequences as herd immunity is eroded, it is imperative to understand the factors that contribute to decision-making about vaccines. Recent scholarship on the concept of “vaccine hesitancy” emphasizes that vaccine behaviors and beliefs tend to fall along a continuum from refusal to acceptance. Most research on hesitancy has focused on parental decision-making about childhood vaccines, but could be extended to explore decision-making related to adult immunization against seasonal influenza. In particular, vaccine hesitancy could be a useful approach to understand the persistence of racial/ethnic disparities between African American and White adults. This study relied on a thematic content analysis of qualitative data, including 12 semi-structured interviews, 9 focus groups (N=90), and 16 in-depth interviews, for a total sample of 118 (N=118) African American and White adults. All data were transcribed and analyzed with Atlas.ti. A coding scheme combining both inductive and deductive codes was utilized to identify themes related to vaccine hesitancy. The study found a continuum of vaccine behavior from never-takers, sometimes-takers, and always-takers, with significant differences between African Americans and Whites.  We compared our findings to the Three Cs: Complacency, Convenience, and Confidence framework. Complacency contributed to low vaccine acceptance with both races.  Among sometimes-takers and always-takers, convenience was often cited as a reason for their behavior, while never-takers of both races were more likely to describe other reasons for non-vaccination, with convenience only a secondary explanation.  However, for African Americans, cost was a barrier.  There were racial differences in trust and confidence that impacted the decision-making process. The framework, though not a natural fit for the data, does provide some insight into the differential sources of hesitancy between these two populations. Complacency and confidence clearly impact vaccine behavior, often more profoundly than convenience, which can contribute either negatively or positively to vaccine acceptance. The Three Cs framework is a useful, but limited tool to understanding racial disparities. Understanding the distinctions in those cultural factors that drive lower vaccine confidence and greater hesitancy among African Americans could lead to more effective communication strategies as well as changes in the delivery of vaccines to increase convenience and passive acceptance.

## Background

In public health, few developments have been as successful as the widespread adoption of vaccines to combat infectious diseases.^1^ Continued success depends on sufficient numbers of individuals receiving vaccines to create and maintain herd immunity. Recently, the challenges posed by vaccine refusal have spurred research interest to understand public attitudes. Vaccine hesitancy has emerged as new concept in immunization scholarship to “de-polarize” the prevailing representation of pro-vaccine and anti-vaccine positions, by portraying the wide range of vaccine attitudes along a continuum from refusal to acceptance.^2^ This new scholarship contributes to the global effort to examine vaccine concerns in different contexts.^3^

In the United States, African American adults are significantly less likely to be immunized for seasonal influenza than White adults.^4^ During the 2015-16 flu season, the Centers for Disease Control and Prevention estimated that 37% of Black adults were vaccinated compared to 45% of white adults.^4^ This disparity has persisted even as influenza immunization rates increased overall with the gap between Black and White remaining nearly constant.^5^ Despite significant research, the specific factors driving this disparity continue to elude scholars. The concept of vaccine hesitancy offers a new lens to explore, and perhaps better elucidate, these persistent racial disparities.

*What is Vaccine Hesitancy?*

The World Health Organization(WHO) defines vaccine hesitancy as;

Delay in acceptance or refusal of vaccines despite availability of vaccination services. Vaccine hesitancy is complex and context specific varying across time, place and vaccines. It includes factors such as complacency, convenience and confidence.^6^

The Strategic Advisory Group of Experts in Immunization (SAGE), a working group of scholars selected by the WHO that is routinely assembled to assess emerging issues related to vaccination, developed the concept.^7^

The WHO definition emphasizes three factors central to vaccine hesitancy, the Three Cs, of complacency, convenience, and confidence.^2^ Complacency refers to how individuals perceive the risk and value of vaccines. Convenience refers to factors associated with access to the vaccine, including availability, affordability, and accessibility. Confidence refers to “trust in the effectiveness and safety of vaccines, the system that delivers them, including the reliability and competence of the health services and health professionals and the motivations of policy-makers who decide on the needed vaccines.”^8^

Additionally, the WHO definition emphasizes the context-specific nature of vaccine hesitancy. For instance, vaccine hesitancy can be specific to a particular vaccine, with individuals or groups delaying or refusing some vaccines but not others.^2^ The SAGE working group outlined a “matrix” of determinants that influence vaccine hesitancy, including contextual influences such as historic, cultural, environmental, and political factors; individual and group influences including personal experiences; and vaccine specific issues.^2^ There are calls for future research to understand dynamics of hesitancy within sub-groups, and to explore the broader social, historical and cultural context from which hesitancy arises.^9^^,^ 10

In 2015, Peretti-Watel and colleagues noted some conceptual ambiguity in vaccine hesitancy.^11^ While many argue that vaccine hesitancy exhibits itself as behaviors along a continuum from complete acceptance of vaccines to total refusal, including delay or refusal, Peretti-Watel and colleagues argue that vaccine hesitancy is a decision-making process, affected by the context in which it is located, and ultimately, affecting behavior itself.^11^ Similarly, Salmon and colleagues highlight the role of underlying beliefs and attitudes in shaping vaccine hesitancy, warning that observed vaccination behaviors are often influenced by external factors and therefore, may not reflect true levels of hesitancy.^12^ As an emerging concept, a majority of published literature on vaccine hesitancy has been largely theoretical. Two recent qualitative studies were among the first to apply these theoretical concepts to an actual dataset. Both studies took a broad view that focused on measurable behaviors but also explored the attitudes that motivated those behaviors.^13^^,^ 14 For our study, we utilize the broader conceptualization of vaccine hesitancy, by measuring vaccine behavior and exploring attitudes related to complacency, convenience, and confidence.

Traditionally, vaccine research has utilized a number of theoretical models including the Health Belief Model, the Theory of Reasoned Action, and the Theory of Planned Behavior (for recent examples, see 15 ^,^ 16 ^,^ 17). These theories have centered on individual attitudes, beliefs and internal thought processes. We see the Three Cs framework as providing broader sensitizing constructs that attempt to incorporate social and contextual factors and potentially capture the wider scope of vaccine hesitancy. While there is some similarity and overlap between constructs from existing theories, most notably the Health Belief Model, these constructs can take on new meanings within the Three Cs framework. Indeed, one of the major critiques of the use of these expectancy-value theories in vaccine research is the limited findings that they produce.^2^

To date, the majority of research on vaccine hesitancy has focused on the role of vaccine hesitancy in parental decision-making related to childhood vaccination.^18^ In the US, a smaller body of work focuses on understanding the role of vaccine hesitancy in adult vaccination.^19^ Several factors may explain this focus, including that the specified timeframe for childhood immunization makes it easier to assess hesitancy and delay, the growing political debate over mandatory vaccination policies, and high-profile outbreaks of vaccine preventable illnesses.^2^^,^ 12 ^,^ 20  However, because the flu vaccine is recommended annually, hesitancy is an issue that can have yearly impact on vaccine acceptance and delay, and since flu vaccine is recommended for all people over 6 months of age, hesitancy can contribute to greater disease burden across the population. This paper is one of the first to qualitatively explore the role of vaccine hesitancy in adult influenza immunization.

Given the consistent disparities in flu vaccination between African Americans and Whites, exploring vaccine hesitancy for its relevance to understanding these disparities is potentially useful. Additionally, little is known about how socio-economic status, race and racism may impact these concepts. This study explores whether vaccine hesitancy and the Three Cs framework is an applicable concept with adults and explicitly explores the differences between African American/Black and White Adults. Our research questions are:

1. What is the difference in the degree of vaccine hesitancy between African American and White adults related to seasonal influenza immunization?
2. What impact do cultural, attitudinal and social differences have on vaccine hesitancy?
3. Are the vaccine narratives of both African American and White adults accurately reflected in the Three Cs framework?

## Methods

*Data Collection*

Utilizing an iterative process of data collection and analysis, we collected our qualitative data as part of a larger mixed-methods investigation of racial disparities in influenza immunization in African American and White adults. Our sample included native-born, English-speaking, adults over the age of eighteen, who identified either as White or African American. About two-thirds were African American and one-third was White. Data collection began with semi-structured exploratory interviews (N=12) to identify factors that influence vaccination decisions for African Americans and Whites. Based on that data, literature review and previous research, we then conducted nine focus groups (N=90), separating groups by race (6 African American, 3 White), vaccination behavior (3 Vaccine Takers, 3 Non-takers, 3 Mixed), and geographic location (6 Urban/suburban, 3 Rural). To facilitate research on racial identity and vaccination beliefs, we intentionally over-sampled for African Americans with purposive sampling to ensure diversity within racial groups for variables such as gender, age, and income. The focus groups followed the “funnel approach” in which moderators followed an open-ended interview protocol and directed the conversation while allowing discussion to flow freely.^27^ In an iterative process, we adapted the protocol based on our results. Based on the focus groups, we identified specific areas for further elaboration in sixteen in-depth, individual interviews. Our total qualitative sample included 118 adults (N=118).

The Institutional Review Board at the University of Maryland approved all data collection activities (367080). The study sought to recruit African American/Black and White adults born in the US and of both genders. The rationale for inclusion of both racial groups was the noted disparity in adult vaccination between African Americans and Whites. Adult was defined as 18 years of age or older. During the screening process, all participants were asked to self-select their race and gender. Each participant received a paper copy of the informed consent request and discussed it with project staff prior to their signature.

**Recruitment Strategies:** 

Phase of data collection	Location	Type of Sample	Recruitment Strategies
Exploratory Interviews (1-1.5 hour)	Washington, DC metropolitan area; Athens, GA	Convenience sample with quotas to ensure appropriate representation by race, gender, age, and vaccine status	Email invitations and flyers sent to local contacts; Email flyers distributed through MD-Community Research Advisory Board; Flyers and visits to local barber and beauty shops associated with the Maryland Center for health Equity (M-CHE); Facebook page for the study; Announcement on the M-CHE webpage; Incentive $30 giftcard
Focus Groups (1.5 hour)	Washington, DC metropolitan area; rural Eastern Shore, MD	Purposive sampling to ensure appropriate representation by race, gender, age, and vaccine status	Advertisements in the Washington Post Metro Express (free daily paper in DC region), The Diamondback (campus newspaper), and in the Star Democrat (a free daily paper in the Eastern Shore region); Email flyers for distribution through the MD-Community Research Advisory Board; Flyers and visits to local barber and beauty shops associated with the M-CHE; Facebook page for the study; Announchement on the M-CHE webpage; Incentive $50 giftcard and refreshments
In-Depth Individual Interviews (1-1.5 hours)	Washington, DC metropolitan area	Purposive sampling	Email invitiations and flyers sent to local contacts; email flyers for distribution through MD-Community Research Advisory Board; Facebook page for the study; Announcement on the M-CHE webpage; Incentive $30 giftcard

**Sample Descriptions:** Exploratory Interviews

	White (N=5)	Black (N=7)	Total (N=12)
Gender (%)			
Female	100%	29%	58%
Male	0%	71%	42%
Age Range (yrs)	27-71	26-65	25-71
Mean Age (yrs)	46.2	41.9	43.8
Flu Vaccine Status (%)			
Yes	83%	43%	54%
No	17%	57%	46%
Education Level (%)			
Less than High School	0%	14%	8%
High School/GED	17%	29%	23%
Some College/Associate Degree	33%	29%	31%
Bachelor's Degree or Higher	50%	29%	38%

**Sample Descriptions:** Focus Groups

	White (N=26)	Black (N=64)	Total (N=90)
Gender (%)			
Female	62%	34%	63%
Male	38%	64%	36%
Other	0%	2%	1%
Age (%)			
18-29	15%	9%	11%
30-44	19%	25%	23%
45-59	12%	42%	33%
60+	54%	23%	32%
Flu Vaccine Status (%0			
Annually	44%	37%	39%
Most Years	15%	18%	17%
Once or Twice	25%	22%	20%
Never	26%	24%	24%
Education			
Less than High School	0%	3%	2%
High School/GED	0%	28%	19%
Some College/Associates Degree	22%	39%	34%
Bachelor's Degree or higher	78%	30%	44%

**Sample Descriptions:** In-depth Interviews

	White (N=8)	Black (N=8)	Total (N=16)
Gender (%)			
Female	50%	50%	50%
Male	50%	50%	50%
Age Range (yrs)	24-67	35-72	24-72
Mean Age (yrs)	44.8	55	49.3
Flu Vaccine Status (%)			
Annually	38%	13%	25%
Most Years	0%	13%	6%
Once or Twice	13%	13%	23%
Never	50%	63%	56%
Education Level (%)			
Less than High School	0%	0%	0%
High School/GED	0%	0%	0%
Some College/ Associate Degree	13%	13%	13%
Bachelor's Degree or Higher	87%	87%	87%

*Data Analysis*

For this manuscript, we conducted a thematic analysis to specifically explore the role of vaccine hesitancy and the Three Cs framework across our sample. Thematic analysis evolved as a method of textual analysis that builds off of a traditional content analysis, but emphasizes the qualitative meaning attached to a text rather than simply documenting the frequency of codes.^21^ All interviews and focus groups were professionally transcribed and imported into Atlas.ti. Two team members coded all transcripts and discursively resolved all coding discrepancies, reaching consensus on each code. The coding process included an initial, line-by-line, coding and later-stage focused coding of broad concepts. In our initial coding process, we began with a codebook of fifty deductive codes as a guide. Examples of deductive codes included “perceived risk of disease” and “barriers to access”. Following a careful line-by-line technique, we either used existing codes or created new codes to fit each idea we observed. Examples of emergent codes included “prefers natural remedies” and “not necessary”. The final codebook included 121 codes. At this point, we recognized similarities between our codebook and the Three Cs framework presented in the vaccine hesitancy literature. To conduct the thematic analysis, we developed a set of deductive codes derived from the Three Cs Framework including codes of “complacency – positive” and “convenience –negative” and recoded our data with these new codes.

Significant themes around each of the Three Cs were fully developed into written memos combining theoretical definitions with illustrative quotes. A major part of thematic analysis is identifying and recognizing patterns in the data.^22^ We utilized a visualization feature on Atlas.TI to understand the interconnections and overlap between major codes. Our thematic approach is perhaps best characterized as a “hybrid approach” as it combines both inductive codes and deductive codes derived from an a priori framework. This is similar to the work done by Fereday and Muir-Cochrane (2006), who argue that the hybrid approach that balances inductive and deductive coding can actually demonstrate greater rigor in qualitative research.^23^

## Results

Our data revealed a continuum of vaccine behavior from never-takers, sometimes-takers, and always-takers with great variability within these groups, especially in motivations and the ways their attitudes have changed over time. By focusing on behavioral outcomes alone, this variability was lost, so we broadened our analysis to focus on the major motivations that drive different vaccination behavior. We made a decision to see how well our findings “fit” into the Three Cs framework. While the WHO definition broadly describes the Cs, no one had yet applied these conceptual definitions to an actual data set of American adults. Since each C had an influence in both directions, with some elements contributing to greater vaccine hesitancy and some attitudes contributing to greater vaccine acceptance, this approach was much “messier” than the literature would suggest. We explore the role of vaccine hesitancy in influenza vaccine disparities by examining articulations between the narratives of our participants and the Three Cs of complacency, convenience and confidence.

To identify quotations, the following four part coding system is used: First, race either W=White or AA=African American, followed by gender F=Female and M=Male, then vaccine status (if known) T=vaccine taker or NT=non-taker. The final code reflects the stage of data collection: EI = Exploratory Interviews, FG=Focus Groups and II= In-depth Interviews.

*Complacency Contributing to Vaccine Hesitancy*

In the SAGE framework, complacency is used to characterize the combination of low perceived risk and low motivation to vaccinate.^8^ Interpreting this definition, we recognized a distinct subset, generally young and majority white, of our sample could be described as complacent. For some participants, complacency was characterized by true apathy. One young white non-taker described it:

I mean there is not really anything that wouldn’t make me want to get it every year, so I feel like I’m kind of in this ambiguous space where I probably should, like there is no reason why I shouldn’t, but it’s just not something I think about. If I thought about it, I would probably do it, but it just has never been a part of my life. So I think I am just conditioned to not even think about it.

(WMNT–II)

The majority of complacent non-takers perceived a low susceptibility and low severity of seasonal influenza as justifications to forego vaccination. Non-takers often described the flu as “*just a bad cold*” or “*not that* *big of a deal*”, especially when compared to more “*serious*” diseases like polio or measles. Some believed that the flu could be serious, but only to individuals who are “*high-risk*” and not people like themselves. One man used his perceived low risk as a deciding factor against a vaccine:

Since I’ve never had the flu and I think of myself as a very healthy person, it just doesn’t matter to me. So when the doctor asked me, "Are you going to get a flu shot?" I said, "No, I don’t plan on it."

(WMNT-FG)

The most common manifestation of complacency was that the belief that the flu vaccine is simply “*unnecessary*.” For non-takers, there were two positions: those who believed that their immune system was fully capable of fighting off infection without a vaccine and those who practiced other behaviors they believed to be more effective in preventing infection. Within the first group, crediting a strong constitution was common - as an African American man explained, “*Over the years, I decided not to take the flu shot, because I feel like I was healthy enough and my immune system was strong enough to avoid the germs or bacteria, whatever that causes the flu*” (AAMNT–FG). Members of this second group attributed their health to “*common sense*” prevention techniques: *“I’ve never had the flu before, even without getting the shot, so apparently I’m doing something right or whatever the other ways, washing my hands. Whatever it is, I’ve never felt the need to get the shot*” (AAFNT–FG). Within both groups, there was a sense that healthy individuals can rely on alternatives to the flu vaccine.

We noted an interaction between complacency and an incomplete understanding of how vaccines work among a small proportion of mostly African Americans respondents. For example, some participants did not understand why they needed a vaccine if they are not sick. One man described, “*I just can’t get around the fact of injecting myself with flu so that I can become protected by the flu*” (AAMNT-II). These misconceptions reflect both fear and doubt, but also contribute to the belief that the vaccine is not necessary.

*Complacency Contributing to Vaccine Acceptance*

Interestingly, what appeared to be complacency did not always lead to non-vaccination; in fact, we saw several instances where it contributed to passive acceptance of the vaccine. One young man described how he got his first flu shot at his physician’s urging, “*I got it because my doctor strongly, strongly encouraged me to. And it was right there and I was right in the doctor’s chair. But I think if I had to make the effort to go out and do it myself, I wouldn’t have gotten it”* (AAMT-II). Another person couldn’t remember the reason for being vaccinated this year, “*I took it last year, but the last few years before that I didn’t… I couldn’t be bothered, figured nothing was going to happen. I’m not sure why I got it this last year*” (WT-FG). More passive vaccine takers didn’t have a strong opinion on the vaccine, but when external circumstances made vaccination an option, they accepted it.

Among our respondents, many did not object to the vaccine; they simply felt that it was not necessary. Under certain circumstances, this translated into vaccine acceptance, but more frequently, it contributed to greater hesitancy. Younger participants displayed higher levels of complacency and apathy. There were no marked differences by gender or race.

*Convenience Contributing to Vaccine Hesitancy*

In the SAGE Framework, convenience refers to availability, affordability, and accessibility of vaccines.^8^ More traditionally, these concepts may be thought of as barriers, but convenience captures both the presence and absence of barriers. However, this is one area where our qualitative data did not match the Cs framework and for many of our participants, convenience and barriers were substantially different concepts. We found that participants tend to consider convenience in conjunction to other factors, and that there were significant differences between takers and non-takers.

Major barriers to vaccination related to affordability included vaccine costs, transportation, and health insurance. Most participants knew how much flu vaccines cost and where they could go to receive them. Several focus groups engaged in heated discussions of the best locations to get low-cost vaccines. Although many recognized the relatively low prices of flu vaccines, some participants felt they were still out of reach for the poorest of the poor. One man explained, “*So it’s like 10, 15 dollars. I do think that if you have children, let’s say you have five kids, $15 a person, that's excessive*” (AAMT-EI). While both racial groups discussed poverty as a barrier to vaccination, African Americans were more likely to describe personal or community experiences of poverty. This was the reality for one African American woman:

I mean for me, it’s challenging-- most people probably don’t have six children. But, the size of my family, and working, and trying to schedule it in, and get all the appointments and all that, you know, I tend to stagger them. And so, if that flu shot is not done in one fell swoop, that requires additional visits to the doctor, which can be really hard to do, especially a single parent.

(AAFNT-EI).

Transportation costs were another barrier for elderly or low-income participants. Another woman explained her transportation difficulties, “*The money to pay for it, transportation for some people, especially like in areas like this, that I'm more rural, a way to get to the doctor, or if somebody is having a free clinic of flu shots, and how are you going to get there?*” (AAFNT-EI). College students also shared this view, explaining that with their very limited budgets (and relatively good health), the vaccine was an unnecessary cost, especially if it meant a trip to the doctor’s office, or a trip “*home*”.

For some, it was a struggle to justify the cost of the vaccine, especially without medical insurance. One older woman who had recently lost her job and insurance described, “*If public healthcare covered it, I would be much more likely to do it, but if I have to budget it in when I’ve been getting it free for several years, it’s different*” (WFT-FG). Another man explained, “*Another deciding factor for me not getting the flu shot is lack of insurance. There were times where I didn’t have insurance*” (AAMNT-FG).

*Convenience Contributing to Vaccine Acceptance*

We observed a division between takers and non-takers related to convenience. Overall, vaccine takers were more likely to cite convenience as a reason they got the vaccine, while implicitly implying the true reason they got the vaccine was obvious – “*it works*”. Non-takers were more likely to cite other reasons, such as personal beliefs or philosophical reasons first, and then describe barriers as a secondary justification. Several non-takers actually described how easy it would be for them to get a vaccine if they wished, but that they had made a decision not to do so.

Takers described accessible location, low cost, and encouragement through workplace policies as the most common convenience-related reasons for vaccination. Widespread availability in pharmacies, grocery stores, and pop-up clinics made the vaccine very accessible, as one woman described:

You know you’re in Costco on a Saturday and they’re there, and you’re like, “Oh I should get my flu shot.” Not that you had planned on it, but like you said, it’s a pain organizing a trip to the doctor. Having it be very convenient makes it easy.

(WFT- FG)

A woman described her preference for the clinics over her doctor’s office, because she could avoid paying additional co-pays and visitation fees, “I find that’s cheaper than going to my doctor. I go to my doctor, I got the office visit, plus I have the cost of the flu shot. If I just go to the local clinic, then I just get the cost of the flu shot" (AAFT-FG).

Workplace policies also have a positive impact on vaccine acceptance. Participants described policies ranging from passive to proactive that made the vaccine more convenient. Most involved small incentives that helped them decide to get a flu shot; one federal intern said she could easily get it in her office building; another office worker described receiving regular e-mail reminders from her boss; and still another said her entire office would all go together to the clinic for support. Other employers relied on more persuasive methods. A university student explained how his supervisor offered to cover the cost. Some employees were required to get their flu vaccines as a condition of employment; this included a hospital nurse, a student health volunteer, and several current or former military personnel.

Conceptually, we found a great deal of overlap between complacency and convenience. For some individuals (especially those with high levels of complacency), convenience may make the difference between vaccinating and non-vaccinating, “*I’ve always been willing to take it. I’ve never had any resistance to taking it. The shift has just been convenience and that the awareness is right there in front of me*” (AAMT-II). Another woman explained:

I’m not opposed to it in anyway, certainly. For example, if my place of work said, “Hey, we’re doing flu vaccines today. Would you like that?” I probably would get one. I don’t have any reason not to. I’m not afraid of it in any way. I just don’t go down to CVS and get it or whatever.

(WMNT- II)

*Confidence Contributing to Vaccine Hesitancy*

The final, and most complex, C is confidence, which the WHO defines as trust in vaccines and the broader system that produces, distributes, and provides them, along with the policy makers responsible for vaccination.^8^ Indeed, as a concept, there is a great deal of overlap between confidence and trust. We found that participants were more likely to use the language of trust to describe their own views– specifically, trust in the vaccine’s effectiveness and safety, as well as trust in the health care system that provides the vaccine and the policy makers who set the vaccine regulations and policy. We observed three major areas related to confidence: trust/distrust of the vaccine, trust/distrust of the agencies that produce the vaccine, and a more generalized sense of trust/distrust that stems from traditions. While many factors were shared across our participants, Whites and African Americans expressed differing perspectives on trust, with Whites demonstrating greater trust, while African Americans were generally more skeptical.

*Vaccine Distrust*

We frequently heard statements like “*There's something about the flu vaccine I don't trust…*” (AAFNT-EI). For these individuals, low vaccine confidence was linked to high levels of distrust in the vaccine itself. While many participants could not articulate exactly why they were hesitant to get the vaccine, some justifications included the fear of side effects or “*bad reactions",* distrust of the vaccine due to low perceived efficacy, belief that the flu vaccine would actually cause influenza, and fear of needles.

A major challenge to vaccine confidence is the fear of side effects, which was widespread across participants. One man described how his fears arose after hearing a list of potential risks:

There was a disclaimer on the flu shot stuff that says you’re subject to, in very rare instances that there is a type of paralysis that can happen and that is basically debilitating… And I thought, ‘Well, you know, I’m not going to take a flu shot.

(WMNT-II)

Another woman described that knowing about potential side effects “*is very important*” in her vaccine decision. When she heard about side effects, she wondered, “*What is the side effect? What else would be triggered in me?*” (AAFNT-II).

These participants may have based their fears on personal experiences or anecdotes of “bad reactions” that they had heard from friends and family members. Some were based on specific instances, such as this young woman’s description of her sister’s experience:

“My sister had a very, very bad experience with a vaccine, so that kind of scares me off a little bit... She had a very bad reaction. I mean she lost a ton of weight. She was down to like 80 pounds. She couldn’t get out of bed for like three months. It was just very, very bad

(WFNT-II)

Others were fueled by rumors of extreme side effects. Respondents were quick to repeat some of the stories they had heard, “*I hear people, I hear tall tales, someone will say, ‘It will paralyze you. They’re injecting a live virus in you and it’s going to kill you. No, no, no, I’m not doing it*” (AAMT-II). One viral internet story about a Washington Redskins cheerleader resonated with African American participants:

There was something a couple years ago. It was like on the news. It was like a Redskins cheerleader where she got the flu shot and she had the same issue where it was like she couldn’t even talk or something was going on with her. And so I never felt like, because I don’t get sick, so it’s like why should I take the chance of getting it and then having something like that happen?

(AAFNT-FG)

Whether the fear of vaccine side effects stems from a personal experience, an account from friends and family, or from a rumor, these fears had a strong, negative impact on trust in the flu vaccine and vaccine confidence overall.

Individuals actively weigh the risk of disease against risk of side effects. For people who already perceive low vaccine benefit and low disease risk, the fear of side effects may be enough to deter them from the vaccine. For instance, a woman explained, “*Given that it’s not totally effective, why would I then want to make myself sick for two days only to be protected from something that probably won’t happen anyway?*” (WNTF-FG). News stories that discuss the actual efficacy of vaccines may have exacerbated these fears: “*You think of it as like 100% and it was, I don’t know, something closer to 50%. I didn’t find that compelling enough to go over to the doctor’s office*” (WFNT-FG).

Some individuals were also afraid that the vaccine would give them the flu. One man elaborated, “*I have heard too many horror stories from people that I know where they took the flu vaccine and they got real ill. I’m like, ‘Okay, that validates my thought right there*” (AAMNT-FG). The indiscriminate use of the term “flu” to describe a variety of ailments makes it difficult to confirm if these people were actually contracting influenza, but the perception that the vaccine caused the illness it was intended to prevent, reduces trust in the vaccine and negatively impacted vaccine confidence. These fears led some individuals to forego vaccination, as this woman explained, “*A friend of mine just got the flu shot and it was like they had the flu. I’m like, 'Oh my goodness, I am never going to get the flu shot.’ And I never have and I don’t think I ever wil* *l"* (AAFNT-FG).

Distrust of vaccines and fear of needles arose among some African American participants. This African American man described both of these fears, “*A lot of things I don’t like about needles is contamination. I don’t really like taking needles for any reason and I don’t like the pain of needles*” (AAMNT-FG)

*Organizational and Governmental Distrust*

Low trust for the organizations involved in vaccine approval, manufacture, and distribution negatively impacted vaccine confidence. We assessed trust in motives as well as trust in the competence of pharmaceutical companies, public health agencies, and “*the governmen* *t*” more broadly.

Distrust in pharmaceutical companies was universal among many participants young and old, male and female, White and African American. Some concerns centered on their ‘for-profit’ nature, as this white woman explained “*I’m not sure the vaccine works. I think it might be a big hype by the pharmaceutical companies to get more money*”(WNTF-II). Another African American man explained, “*You're not really concerned about me as much as you are about your financial bottom line*”(AAFNT-EI). Individuals questioned not only the motives of pharmaceutical companies, but also their ability to produce safe, effective, and reliable products:

So, it's like you're asking me to trust a pharmaceutical company when anybody can do anything to that. I'm swallowing, I'm injected, I'm taking it because it's a pharmaceutical company and that's it? What kind of oversight are we talking about here?

(AAFNT-FG)

Some participants discussed their distrust of public health agencies, but these sentiments were more nuanced. One woman described her ambivalence, *“I honestly don’t know how the FDA can test to make sure that the vaccine works, but I do trust that they are testing it to make sure that it is safe*” (WTF-II). White participants voiced concern about the competence of these agencies, but among some African Americans, concerns related to motives that drive these agencies. When asked to describe why he has “*zero trust*” in the people who produce the flu vaccine, this man explained, “*Because, I mean, why would they really care so much about us?*” (AAMNT-FG).

The clearest racial divide in vaccine confidence was between White and African American participants’ different levels of trust in the government’s role in vaccination. White participants expressed greater trust in government, while African American participants voiced lower trust, with particular concerns regarding the government’s motives. A common refrain from black focus groups was, “*You don’t trust a government vaccine*” or “*don’t trust the government for nothing*” (AAFNT-FG). This distrust extended into conspiracy theories including beliefs that the government was experimenting on minorities as “*guinea pigs*”, that the vaccines were being diluted and distributed in Black communities, or that vaccines were a form of population control. Additionally, the legacy of the Tuskegee Syphilis Study emerged in every focus group as a justification for distrust.

Our findings revealed a significant influence of family traditions on vaccine confidence. In the absence of a strong family tradition supporting vaccination, many individuals, especially from older generations, refused to consider a flu shot now. Another older woman explained:

Only because I grew up with my mother, grandmother, father, great-grandmother, everybody around me, "No, I'm not taking that. It ain't gonna give me the flu." I mean, that's always what I've heard, so when I became an adult, "Well no, not going to give me the flu either.’"So huh-uh, don’t do it.

(AAF-FG).

This is especially true among African American families, where distrust of the medical establishment was the norm, as this older man explained, “*I was raised old school, so a flu shot was not part of the regime to be taken care of, and then there was a time where the flu shot was killing people. There were counterfeits*” (AAMNT-FG). In many instances, the confluence of distrust in the vaccine, distrust of the medical establishment, and a lifetime of socially reinforced fear of the vaccine, reinforced very low levels of vaccine confidence among African Americans.

*Confidence Contributing to Vaccine Acceptance*

Individuals who regularly get immunized for flu described having greater confidence in flu vaccine. This stemmed from higher levels of trust in the vaccine, as well as trust towards public health and government authorities responsible for producing vaccine, and health care providers administering it.

People who trusted the vaccine often described it in terms of vaccine effectiveness. Many takers got the vaccine because they believed it works. “*I believe in vaccinations*” is how one man succinctly described his support (WMT-FG). These participants also viewed vaccines as the primary method to prevent disease. When asked how she prevents flu, a woman answered, “*What comes to my mind is get the flu shot...*” (AAFT-FG). An older man was declarative, “*I keep up on my shots, and I believe in the system… I mean I have not had the flu. I really don’t get sick and I think it’s because of the vaccine. And so I kind of believe it, I really do*”(WMT-FG).

Vaccine takers also weighed perceived risks and perceived benefits of the vaccine, but ultimately decided in favor of the vaccine. One difference was that African Americans considered rare and extreme risks from the vaccine while Whites focused on more minor risks and inconveniences. A man who admitted that he only sometimes got a flu vaccine described this weighting of risks and benefits among people in his community:

So they’re [his non-vaccinating friends] always thinking about okay, it [vaccine safety] might be 99%. They’re thinking I could be that 1% that gets sick, dies. That’s the only thing I could do. In other cases, another person might look at it, "Well, 99%, that’s better than 50% chance you getting it if you don’t take this vaccine."

(AAMNT-EI)

For some, ambivalence created an overlap between confidence and complacency. This was captured in a few "shrugging" type comments where people felt the vaccine might help and would probably not harm them. For some White takers, the comparison of risk was less extreme, as one woman explained, “*Well, it’s the lesser of two evils, the way I look at it… I've had no side effects, knock on wood I've been healthy for all these years in spite of taking it or because of taking it*” (WFT-EI). Whites also perceived very few risks to the vaccine, so that a risk comparison was almost one-sided: “*But I just feel like if it’s not hurting you, you might as well try it and maybe it will help you or maybe it will prevent something*” (WFT-EI).

For Whites, the power of family traditions and the intergenerational transfer of health practices emerged as significant source of vaccine confidence whereas for many African Americans, traditional family beliefs diminished reduced vaccine confidence.

*Organizational Trust and Governmental Trust*

For non-takers, a lack of trust in any one area (i.e. vaccine, pharmaceutical companies, federal regulatory agencies, the government) was often enough to reduce vaccine confidence. For takers, high confidence reflected trust in the overall process. These individuals describe trust very broadly. A young woman described her attitude as, “*I trust the product and trust the people*” (WFT-II). Similarly, an older man explained the many facets of trust:

Well, it means I trust that the vaccine is going to be effective, I trust that nothing dangerous is being given to me, and I trust the sources of the vaccine, meaning, I mean that’s a lot of trust, but I’m trusting the makers of the vaccine, I’m trusting my doctor who recommends it, and I’m trusting the U.S. government who promotes it and subsidizes it to some extent. So it is a lot of trust. And I think if any of those factors were not in place, I would probably have some doubts about the vaccine and may or may not take it, so trust is key.

(AAMT-II)

We also observed differences between individuals who voiced an unquestioning and implicit trust in vaccines and others who had reached a high level of vaccine confidence after much consideration and debate. Both groups had high vaccine confidence but it stemmed from different origins, falling along racial lines. Whites were more likely to simply ‘trust’ a vaccine while African Americans were more likely to engage in thoughtful discussion. One woman was asked why she had listed “trust” as unimportant in relation to her vaccine decisions: “*I’m a trusting person. That’s why it’s so unimportant. Inherently, it’s there*” (WFT-II). For the African American vaccine takers, they were aware of the concerns within their community and yet they made an active choice to trust. One woman described her new approach to healthcare; she saw how distrust had led to her mother’s early death at age 38, so she made a choice to get involved and take a more active role in her own health, “*I don’t distrust government or distrust medical professionals. I do ask questions. You ask questions of more than one person, but you don’t come with the premise of distrust*” (AAFT-II).

**Summary of Themes Organized by Construct:** 

Constructs	Non-Takers	Takers
Complacency	Vaccine is not necessary; Natural immune response is sufficient; Other behaviors prevent flu; Low perceived susceptibility to flu.	Doctor's recommendation overcomes ambivalence towards vaccine.
Convenience	Vaccine (and related costs) are too expensive; Difficult to obtain without insurance.	Convenient location made it easy; Low cost/free; Encouraged through workplace policies; Insurance coverage.
Confidence	Fear of vaccine side effects; Distrust vaccine; Distrust organizations that produce vaccine; Distrust in government; Family history of distrust; Fear of needles.	Trust vaccine; Trust government; Trust organizations that produce vaccine; Family history of trust.

## Discussion

Our research revealed a continuum of vaccine attitudes and behaviors across both White and African American participants. In many ways, this continuum is reflected in the Vaccine Hesitancy Three Cs framework, but there are significant points of deviation.

To some extent, the degree of hesitancy differed by race just as the reasons for hesitancy about the flu vaccine varied by race. Among the non-takers, racial differences were observed based on trust and confidence, complacency, perceived risk of influenza and other factors. However, many white non-takers did not outright reject the flu vaccine, but did not believe they needed the vaccine due to low perceived susceptibility. For the sometimes-takers, external motivations included outside factors like a doctor’s recommendation or a convenient opportunity, while their own passivity contributed to their vaccination decisions as well. The always-takers demonstrated high confidence in the vaccine. For both African Americans and Whites, complacency reduced likelihood of vaccination. For many, complacency was linked to low perceived risk for influenza. For others, complacency intersected with confidence when their low perceived disease risk, coupled with questions about vaccine efficacy, contributed to failure to get the vaccine.

For numerous takers of both races, convenience was cited as a major facilitator of vaccination whereas non-takers cited other reasons for lack of vaccination first, only raising convenience issues as a secondary reason. To overcome complacency, external motivators, including increased convenience, appeared to be important prompts to action for sometimes-takers. In many ways, this reflects what Nichter described as the difference between “passive acceptance” and “active demand” for a vaccine, a concept that is beginning to be integrated into the vaccine hesitancy literature.^9^^,^ 24 These findings suggest that increasing access to affordable vaccine through convenient locations can potentially increase passive acceptance of the vaccine for sometimes-takers.

It was in the context of confidence that racial differences emerged more starkly and cultural, social, historical and attitudinal factors had the most significant impact. Distrust of pharmaceutical companies cut across races, with even takers voicing doubts about pharmaceutical companies. However, in terms of trust in government, Whites typically expressed more trust, and when they voiced distrust, they raised questions about government’s competence. For African Americans, distrust was stronger and specifically coupled with concerns about the government’s motives with regard to racial and ethnic minorities. Increasing trust in the motives of agencies or industry is a longer-term and complex challenge. However, both public health professionals and health care providers can emphasize the primary motive driving promotion of the flu vaccine as the critical importance of protecting individual and public health, and potentially addressing the ‘elephant in the room’ by emphasizing that flu vaccination is not a high profit enterprise for pharmaceutical companies.

Among always-takers, there were fewer racial differences as both shared confidence in the vaccine’s safety and its importance. Although they were similar in their reasons for vaccination, they differed in their decision-making process. White takers were passive in their decision making process, and when they did consider side effects in their risk-benefit comparison, they saw vaccine side effects as minimal. African Americans were more deliberate in their risk-benefit analysis, considering more serious potential risks of the vaccine and weighing the decision carefully. Therefore, those African Americans who did take the vaccine had decided to actively confront and overcome many of the more negative perceptions that are prevalent in the black community, including historical distrust. Although that suggests more possibility for hesitancy and delay, they ultimately accepted the vaccine.

This position reflects what Velan and colleagues have identified as the importance of reflexive processes in the ways individuals manage risk and interpret vaccine-related uncertainty.^25^ They argue that individuals bear a strong “personal responsibility” for managing an overwhelming number of risks, and are forced to be selective about which risks require attention at any given time.^25^ Consequently, by attending to the differences in decision making that we found between African Americans and Whites, public health professionals may more effectively communicate information that addresses not just their judgments about disease and vaccine risk, but may also influence some of the family networks that appear to be important in establishing vaccine attitudes and behaviors. This may be particularly critical among the sometimes takers that could be moved from significant hesitancy toward greater vaccine acceptance.

This article was one of the first attempts to use the Vaccine Hesitancy framework with data on adults. Our qualitative findings yielding a broad continuum of behaviors and beliefs that was unwieldy and not easily distilled into discreet categories based on either behavior or belief. In practice, the Three Cs can reflect some of the complexity but the wide range of variability between populations and the overlap between the Three Cs leaves room for debate and growth. Some would note the potential overlap with key concepts such as susceptibility, severity and barriers from the Health Belief Model. We found a number of factors that are associated with complacency, and in the HBM framework, would be described as low perceived susceptibility and low perceived severity. However, by considering the Three Cs, we also discover passive placement of vaccine opportunities in the path of some complacent individuals, results in vaccine uptake. More traditionally, a HBM approach would lead us to target changing individual susceptibility and severity, which is more difficult, and potentially failing to address vaccine opportunities and convenience.

The potential lack of clarity between the concepts of “confidence” and trust raises questions as well. However, our data helped us make that distinction more clearly. The Oxford English Dictionary defines confidence as “The feeling or belief that one can have faith in or rely on someone or something”.^26^ Clearly, the extent to which the public demonstrates trust in the vaccine process, the relevant organizations, and finally the vaccine itself can contribute to a broader confidence or belief in vaccination, and ultimately, decrease hesitancy. This is a area in which there is still significant work to be done, particularly with African Americans.

One of the intriguing opportunities presented by using the Three Cs framework is the recognition of the complexity of the individual, social, cultural and environmental factors that affect complacency, convenience and confidence. By considering some of that interaction, it gives more opportunity to craft more nuanced messages that move sometimes-takers along the continuum to more routine acceptance. Equally important is that the recognition of the complex interaction of these factors facilitates more comprehensive approaches to reducing vaccine disparities, including those that recognize the importance of families, environment, and policies.

## Conclusion

To address vaccine hesitancy among African American and White adults, the Three Cs framework of complacency, convenience and confidence provides some guidance for more nuanced approaches to overcoming complacency, fostering convenience, and strengthening confidence in each group. Incorporating this knowledge into more tailored communication messages and campaigns, as well as into actual delivery of vaccine, could reduce disparities in vaccine uptake and subsequently prevent morbidity and mortality.

## Competing Interests

The authors have declared that no competing interests exist.

## Corresponding Author

Sandra Crouse Quinn: scquinn@umd.edu

Phone: (301) 405-8825

## S1 Appendix: Data Availability

The following Memo details the recoding scheme used to complete thematic analysis and contains the full set of recorded quotes. The Three C framework and classifications were derived from: Larson, H.J., Schulz, W.S., Tucker, J.D., & Smith, D.M.D. (2015) Measuring Vaccine Confidence: Introducing a Global Vaccine Confidence Index. PLoS Currents, 25(7). doi: 10.1371/currents.outbreaks.ce0f6177bc97332602a8e3fe7d7f7cc

**C: Negative Complacency**

Individuals who quite simply feel that the shot is “unnecessary”:

*I’m saying it’s not necessary for me. I’m not saying it’s bad for me. I’m saying it’s not necessary. I think for me it’s a better word, necessary (AAFNT-FG).*

*And I’m not saying that the flu vaccine doesn’t have any redeeming qualities. I’m not saying it’s bad. Personally for me I just choose not to after that one time. It’s enough going on in my body. (AAFNT-FG).*

*Yeah, before being employed there, I normally do not get the flu shot. And I'm not really for-- I don't really see the benefit for the flu shot, for me personally (WFT–EI).*

*And they’ll say, “Well, I understand, but most people want the flu shot. It works with most people.” I said, “I’m aware that it does, but I’m not most people. I’m me.” Because it’s all about me. And necessary to prevent the flu, I don’t think that is frequently the case (WMNT-II).*

Some see other forms of prevention rendering the shot unnecessary:

*I just feel like that I’m not working at the time, so over the winter I wasn’t or whenever the flu outbreak I wasn’t out every day or out, so I wasn’t exposed. If I was out in the workplace and here and there my exposure rate would be different. Then I would decide. Maybe I would change my mind, but it’s nothing I think about. I think there are effective ways to try not to catch the flu. Like I said, washing your hands, watch where you put your hands, keep them away from your mouth, your face, your eyes, your nose (AAFNT–FG).*

*I’m just not for it. If I felt like I wasn’t a healthy person, I didn’t eat well and do all the things that keep disease away, if I had some kind of a disease that made me more susceptible to getting flu or these awful things then I would maybe opt for the vaccination, but I feel like I could survive it (WFNT-FG).*

*Kind of, and I’ve never had to get the flu-- I’ve never had the flu before, even without getting the shot, so apparently I’m doing something right or whatever the other ways, washing my hands. Whatever it is, I’ve never felt the need to get the shot (AAFNT-FG).*

Others are weighing the risks - but rule in the end, that the shot is still unnecessary:

*I don’t know anyone personally who has died from it, who has had an adverse reaction. I hear coworkers too have said that, but so for me it’s less about how bad the flu shot is. It’s about I don’t think it’s necessary, kind of what you were saying, what’s the risk where if I don’t take it I could or could not get it and if I do take it I could or could not get it (AAFNT–FG).*

*I just think it’s a bad cold, like a really strong bad cold, a stronger version of a bad cold, but you can’t really treat a cold and you probably can’t really treat the flu. You can get a vaccine, I guess, that lowers your risk of getting the flu, but once you have the flu, just like a cold, there is really nothing you can do (WMNT-FG).*

*I’ve heard that the vaccine that was used for this year and past year were very effective. I haven't had a flu shot in about two years. I have had, you know, every now and then. But I didn’t this year. I'm just taking a chance. I've had the opportunity. It doesn’t cost me anything. You know, my insurance, it’s covered. But I just haven't taken the initiative to have one. (AAMNT-FG).*

Some individuals were even more passive about vaccination:

*I mean there is not really anything that wouldn’t make me want to get it every year, so I feel like I’m kind of in this ambiguous space where I probably should, like there is no reason why I shouldn’t, but it’s just not something I think about. If I thought about it I would probably do it, but it just has never been a part of my life. So I think I am just conditioned to not even think about it. (WMNT–II).*

*No, not really. Flu experiences, no, I don’t know anybody that has really had it that bad, and other than just being uncomfortable for a week or so. Likelihood of getting the flu, I don’t care. If I get it I get it. I don’t care. (WFNT-II).*

*Well, clearly I don’t give it a lot of thought when it comes to the flu vaccine, because again it keeps coming back to the fact that I haven’t got it. (WMNT–II).*

*I have, because I know a lot of healthcare workers and most of them through the course of their work have to do so, like nurses and such, so I have certainly considered it. But I don’t know. The time just goes and the next thing you know it’s whatever, well into the flu season and I haven’t got it.(WMNT-II).*

*But this year it honestly just didn’t even-- I think, I feel like I get it every other year, I get the shot every other year, and I feel like it’s almost like I get scared on the second-- I get the shot and I’m like, “Okay, I’m good.” And then I don’t get the flu, so then the next year I’m like, “Oh I didn’t get it.” It’s not on my mind. But I don’t know if in the next year I’ll get it. (WFNT-FG)*

There are the people that just don’t get it. Don’t think about it. Don’t get it:

*Q: Right, okay. Do you think you'll get it in the future? A: You know, I'm going 28 years of not having it. I'm hoping I can make it another 30 years so hopefully not. (AAMNT- EI)*

*MOD: Are you actively thinking about how not to get the flu or was it just not an issue last fall for you? FEMALE: Not an issue. (AAFNT-FG)*

*FEMALE: The only shot I’ve taken when I was a little girl in elementary school, that’s the only shots I’m going to get. I ain’t ever thought about the flu shot, because I just don’t get them. MOD: You just don’t get them? FEMALE: Don’t get them. (AANTF-FG)*

**C: Positive Complacency**

These are people who get the shot, they think it is necessary, but they don’t really question their acceptance of the shot. It is just simply something that they do. (There aren’t many instances where people explicitly state their opinions in this way but I suspect that is because we didn’t really probe patients who did get the shot):

*I took it last year, but the last few years before that I didn’t. It just wasn’t convenient. I was traveling or something couldn’t be bothe* *red, figured nothing was going to happen. I’m not sure why I got it this last year. (WFT-FG)*

*I got it because my doctor strongly, strongly encouraged me to. And it was right there and I was right in the doctor’s chair. But I think if I had to make the effort to go out and do it myself, I wouldn’t have gotten it. (AAMT–EI)*

*And I kept on putting it off, changing the subject because he would say, “Oh, have you gotten your vaccine?” “Oh yeah, well I'm going to get it. Oh yeah, well, I'm going to get it on the way to work. I'll get it this weekend.” And then he finally said, “Okay, get it before you come into work.” Boom. And so yeah, I went to the health center and I got it. And he said, “I will pay for it.” So that's when I first got it. (AAMT-EI)*

*A: I’m just thinking like I’m definitely more, I’m very open to just getting, I don’t want to say getting vaccines in general, that sounds like I’m willy-nilly, like “Stab me with things” but like I got the Gardasil shot in college when it was pretty new, and it was the same kind of thing where it protects against certain strains but not all the strains. But I just feel like, I kind of feel like if it’s not hurting you, you might as well try it and maybe it will help you or maybe it will prevent something.(WFT-II)*

*My general practitioner does recommend it, so I do get it, and this year she says, “Oh let’s just go ahead and do it” so I do try to get it every year, but there is a couple years, one year I did miss, I know. (AAFT-II)*

**C: Negative Convenience**

People who do or do not get the shot primarily due to access, availability, and convenience. People are more likely to cite convenience as the primary reason they got the shot, while those who do not get are more likely to cite other reasons first, and then to follow up with inconvenience as a secondary justification. There is a good bit of overlap between the people who view the shot as inconvenient and the extreme passive complacency people who don’t think it is necessary, or don’t even stop to think about if it is necessary:

*I think I said this to Leah that I was under contract for about seven years with a federal government agency, and when they tightened security they pulled all of the contractors’ badges for reinvestigation, and when they pulled my badge I was no longer eligible for care at their medical services. So I didn’t get my flu vaccine as a result. (WFNT–FG)*

*I priced it at my local government healthcare and they were charging, so I did want to bring that up in this kind of forum that if public healthcare covered it I would be much more likely to do it, but if I have to budget it in when I’ve been getting it free for several years it’s different. (WFNT–FG)*

*When you’re pregnant you’re going to the doctor all the time, but in other years I would catch it some years and I would get the vaccination some years, and other years I might have just skipped it, because I didn’t happen to run into a very convenient opportunity. (WFT-FG).*

*Another deciding factor for me not getting the flu shot is lack of insurance. There were times where I didn’t have insurance. My company, it was either way too expensive and I said, “You know what, I’ll just deal.” And then when I had the insurance it was like, “Okay, maybe I will get the flu shot. Oh we’re out. Oh people are dying. Oh this, that, and the third.” It’s like, “Okay, you know what, never mind.” (AAMNT–FG).*

*I mean I would get it every year. I didn’t know that you can go and get it for free or it’s more accessible for people to use my health insurance. I mean I think I called CVS and they said, “Well, we don’t take your health insurance.” So they sent me the place in Annapolis and I live in Denton and I work in Bowie, so it’s like I, you know. (WFNT-FG)*

*I’m not opposed to it in anyway, certainly. Like for example if my place of work said, “Hey we’re doing flu vaccines today. Would you like that?” I probably would get one. I don’t have any reason not to. I’m not afraid of it in any way. I just don’t go down to CVS and get it or whatever. (WMNT-II).*

One instance of a vaccine shortage:

*FEMALE: Well, for the vaccine actually I was going to get-- I have a 14 year old and she has asthma and I was going to get her the flu shot this year, just because it was so bad and there were so many people getting the flu, so I did think about it, but I didn’t end up doing it. MOD: Tell me why not. FEMALE: Actually her doctor was out of it. They were out of it when I called her doctor’s office, so then I just never pursued it any further. (AAFNT-FG).*

**C: Positive Convenience**

*Q: Do you think since you’ve had it, do you think that you’ll get a flu vaccine? A: From now on, yes. Q: From now on. [laughter] A: Yes. [laughter] Because they’re free at the VA. (AAMT-E*I).

*I got the vaccine, I want to say, maybe the end of September, beginning of October, right around when it first was available to get the vaccine. And there was a clinic. I'm a Kaiser patient so they have like this great clinic that you just go to and get all your vaccines. It’s really nice. It's really,* *really nice. (WFT–EI).*

*It got a lot easier once the drugstores all started offering shots at any time, so like this year I didn’t do it but one time, you know you’re in Costco on a Saturday and they’re there, and you’re like, “Oh I should get my flu shot.” Not that you had planned on it, but like you said it’s a pain organizing a trip to the doctor. Having it be very convenient makes it easy. (WFT-FG).*

*My insurance held a clinic right down the street from where I live, so I just went in that day that they held the clinic for the flu shots. (WMT–FG).*

*The last three years I have, but this year I’ve been interning with the federal government, so they had it for free in my building, which was easier. (WFT-FG).*

*I took it the year prior because CVS made it so convenient. They had those mini clinics, but I had to go to Annapolis and I wish they had it over here, and then they just took my insurance. (WFT-FG).*

**C: Negative Confidence**

Uncertainty and Risks of Vaccine or perhaps questioning the vaccine itself:

*I myself was planning on going to get it, but I didn’t end up getting it because I was really unsure. I was really unsure. It was offered in my neighborhood. Like I said, I live in a nice area and they have it. The CVS they offer it for like $27 bucks. (AAMNT-EI).*

*It’s more geared towards, because it’s something that they do not really know exactly what it is.* *That’s the biggest thing. They hear a lot of stories going out that people are getting badly sick. Some bad effects and some people have died over a flu vaccine. So they’re always thinking about okay, it might be 99%. They’re thinking I could be that 1% that gets sick, dies. That’s the only thing I could do. (AAMNT-EI).*

*First thing that comes to my mind is this - I’m real tight with my doctor and she usually calls me in on it, but when she calls me in on it I’m not sick at that time, so I know that she’s being proactive about it. Now, I’m the type of guy that conspiracy, you know what I mean? Am I going to catch it? Do I need to put a vaccine that is toxic in my body to fight what’s coming? Those are the kind of things in my mind that I’m uncomfortable with when it comes to that. But with the relationship I have with my doctor she tells me what I need because of my physical conditioning. So, it’s a lot of doubt in my mind, is it good or is it not good, do I need it if I’m not sick, will it make me sick if I take it. I just don’t, because they’re giving you the virus. The flu shot, they inject you with the virus. I’m afraid you’re going to give me the virus, so I won’t get it. If I’m going to get it I’ll take my chances. I’ve been doing it for as long as I’ve been living. (AAFNT-FG).*

*Just like I said, I don’t see giving me the virus so I won’t get the virus. That’s my thought on it. (AAFNT-FG).*

*But after looking at the CDC website, I read the entire FAQs, when they say it’s a live virus or whatever it is I’m just not convinced that I want that in my body. Why would I put that in my body? It’s like the risk seems so low to me and I’m not in the targeted population. I just don’t want to put that in my body, a live virus in my body (WMNT-FG).*

*Because there was so much talk about the different strains that were out there and news reports about the percentage of the flu that was actually, the percentage of strains of the flu that actually would be affected by the vaccine or prevented by the vaccine was so small relative to-- You know you think of it as like 100% and it was, I don’t know, something closer to 50%. It just didn’t seem like-- I didn’t find that compelling enough to go over to the doctor’s office (WFNT-FG).*

*I don’t believe in it either. That’s why I don’t take it. (AAFNT-FG)*

*And the main reason is because I know when they do these studies on medicines they have a line where only this many people got liver damage, so we can pass it. Well, I don’t care if it was two. And I know that’s how they release, FDA releases things, even though people got sick or died or whatever. If the number didn’t reach the threshold they set then it’s safe for us to take. (AAFNT-FG).*

*And I don’t know, all of this new stuff is coming out, these new vaccines every year. It’s like you don’t know what, we don’t know what those things are going to do to us. (WNT-FG).*

*I'll put it this way. I wouldn’t take it either if the doctor didn’t tell me I had to take it. Anybody that have pneumonia or any kind of bronchitis three or more times, they got to get it every year automatically. (AAMT-FG).*

Some people question the efficacy of the vaccine:

*I heard people getting sick. They say they got sick, but they say not really getting sick. That he’s like showing body how to fight off the flu. But you know this is a trend—I don’t know how effective it is. They say it’s 50%, I don’t know. I never took it before (AAMNT–EI).*

*I’m not that convinced that the flu vaccinating is always that effective, so sort of that combined (WFT-FG).*

*I’m in a university setting and there are lots of people around who are always sick. And the nurse said something about how it could cause, you could get real sick like two days after you take it or something where the vaccine makes you very sick, and so I was like, “Absolutely not. I’m definitely not doing that.” Given that it’s not totally effective, why would I then want to make myself sick for two days only to be protected from something that probably won’t happen anyway? (WFNT-FG).*

People who fear side effects, worry about "bad reactions" or fear needles:

T*he flu shot makes-- I took it back in 2000 and I haven’t taken it once since, because it made me* *so sick. (AAFNT-FG).*

*FEMALE: Well, when I got it I had to leave work because it made me feel achy and like I had the flu. I took the pneumonia shot and it didn’t bother me. MOD: And you said this was in 2000? Have you had a flu shot-- FEMALE: I haven’t had one since. But I felt the same way. I stopped taking the shots, I stopped taking them because I didn’t feel good and I just felt like I was just getting sick every time I took that. Every time I took the shot, I was sick, you know. So eventually, I start going to this doctor that I'm going to now and it’s a big difference. (AANT-FG).*

*I actually had the flu once in college. And it laid me out. I was, I don’t know, 17, 18 years old. And I could barely get out of bed. In fact, I couldn’t get out of bed. And I just laid there for about three days, where I was just totally incapacitated. And I didn’t take the flu shot for many years, up until probably about 15 years ago. I had a boss who marched everybody in the office down to get the flu shot, because he was like, “I don’t want any of you sick, because I need all of you working for me.” (AAFT-FG).*

*I have no idea. I really don’t. Because I don’t remember but surely I had flu vaccines before that one that went bad, and they were okay, and so I thought, it’s been going on for over two decades and I have not had a flu shot, but I really haven’t had the flu, so maybe I am gambling or dodging a bullet, but it’s worked so far (WMNTM –II).*

*Well, like I said before, I really hate needles. I will not get a shot unless I absolutely have to have it. So, that is one of the main reasons I don’t get it (WFNTF-II).*

*No, I don’t think it was immediately following, but it was like maybe two weeks later. I don’t know how you’re defining immediate, but it wasn’t like two days later I got the flu. I’m pretty sure it was like a couple weeks later I got the flu. But I made the self-assessment that perhaps it was due because I was-- I believe it’s a live virus that is injected into you, and I got pretty sick too. So that was the other variable, that I got very, very sick. I was sick for like three weeks.(AAMNT–II).*

Heard about other people having a bad reaction:

*I still never took the vaccine because when I was in my early teens, I heard about this woman who had vaccine, and it crippled her for life. And it just made her basically into a vegetable. She went into a coma. And I just say [00:04:37]. I don't know what they're doing or how they improved it, but I'm not going to take a flu shot ever. (AAFNT–EI).*

*I’m still going to take my chances, because I think, like Karen was saying, I’ve seen so many people who have reactions to the vaccination that could be more serious than when you come down with the flu. The flu basically is an extension of a common cold that lasts a little bit longer than the common cold. So, what you would do for your common cold for the flu, and you get rid of it. (AAFNT-FG).*

*I know there was something a couple years ago, it was like on the news. It was like a Redskins cheerleader where she got the flu shot and she had the same issue where it was like she couldn’t even talk or something was going on with her. And so I never felt like, because I don’t get sick, so it’s like why should I take the chance of getting it and then having something like that happen? (AAFNT-FG).*

*A friend of mine just got the flu shot and it was like they had the flu. Oh man. I’m like, “Oh my goodness I am never going to get the flu shot.” And I never have and I don’t think I ever will. (AAFNT-FG).*

*I can only go by what I’ve seen people that actually have gotten the shot and what happens to them. Other than that I don’t actually know, because I haven’t taken it, but just to see most of them when they get it they get sick, it deters me from taking it. So I haven’t even tried even going about it. (AAFNT-FG).*

*But anything else that they come out with no I don’t trust it, because I used to work in a doctor’s office and everyone that had the flu shot, I don’t say they got the flu, but the shot made them sick. (AAFNT-FG).*

*And then once before I guess a couple of years ago the cheerleader took it, and how she got sick. I mean she was paralyzed. So I don’t want to put anything in my body that they really don’t know if it will interact with my DNA or what I have going on with my body. (AAFNT-FG).*

*So, I just don’t trust the-- I don’t see the benefit of it. I have friends that get it every year, and they get the flu every year, they get sick. They get sick. They get sick. So I’m like, okay, I’m not taking it and I’ve never had the flu. I did take it one time, six years ago I did. (AAFNT-II).*

*My sister had a very, very bad experience with a vaccine, so that kind of scares me off a little bit. Q: Sure. What was her experience? A: She is a nurse, so they have to take all kinds of vaccines in hospitals. She had the, I think it was the Hepatitis B vaccine. She had a very bad reaction. I mean she lost a ton of weight. She was down to like 80 pounds. She couldn’t get out of bed for like three months. It was just very, very bad. (WFNT-II).*

Do not trust the government's role in vaccine development and production:

*FEMALE: No, I’m just saying in this particular focus group, because of who the Tuskegee experiment involved, wouldn’t that pretty much answer your question right there, wouldn’t that generational scare people for years? MOD: I think for some that is an important topic that has to come up in the discussion. Tell me more about your realization of the connection between the Tuskegee syphilis study and flu vaccination. FEMALE: You don’t get a government issued vaccine. FEMALE: You don’t know what you’re getting. FEMALE: You have no idea what you’re getting. (AAFNT-FG).*

*As far as their chemicals or the people that work for them that are mixing this ingredients together and testing us, because when I was growing up I heard about that Tuskegee experiment, so from that I have never taken anything except for that vaccination that we had to have when we were in elementary school and that was it. (AAFNT-FG).*

*If they were testing people and saying, “Okay, let’s see if your blood will interact with this drug here in the laboratory” then that will be fine. But if they’re just mixing up a bunch of stuff and giving it to everybody no. (AAFMT-FG)*

*Well, I've never went to try to get it. But I'm always told, you know, there's something behind that free. It’s not completely free (AAFNT-FG).*

**C: Positive Confidence**

Weigh risks and favor vaccines:

*About the vaccine, not that-- it’s just that you should get it. I know there's talk about vaccines can harm you. I mean, you are given a strain of a virus when you actually get a vaccine, but it’s-- there are people that have died, you know. But I think it does more good than harm just in general. So not anything negative, it’s just more that you should get it. (AAMT–EI).*

*More people see that it’s-- even though there are horror stories, you feel-- like, I mean, I feel like crap after I get it. That's why I'm not excited to get it. Like, I don’t feel well, like I need to lay down or whatever. I have a slight headache and I feel kind of weakened a little bit. (AAMT-EI).*

*I think in the last analysis it’s a matter of the type of and amount of risk you want to take with regard to making the decision as to whether or not to get this type of vaccine or this type of shot or whatever it is. It’s a personal decision as to how much type of risk you’re willing to take, given all the information you’ve accumulated up to a certain point. (WMT–FG).*

*You can't force them to do anything. I wouldn’t want to force anybody, because I wouldn’t want it forced on me. Usually myself, I do it, I have. But basically, I don’t. And I don’t have any real down to earth reason, other than the needle. The needle was not even an excuse now, because they have the nasal. So I probably will start getting. And the needles are shorter. I've seen them. They advertise them on TV. So that were the motivators for me to do it. And it gives me a sounding board for when that conversation come up with someone, that the needles are shorter now. (AAMT-FG).*

Positive experiences with vaccine, trust the vaccine, vaccine is effective:

*My experience with the flu is I’ve gotten a flu shot every year for 10 years now and the reason is I caught a really bad case of the flu right before Christmas, of course, which is usually when these sort of things can happen. (WFT–FG).*

*A: I didn’t have the sniffles and take the cough syrup and all that, then it go away. But this time it just stayed, and stayed, and stayed; wouldn’t leave. So I figured I better go find out what this is. Q: Do you think since you’ve had it, do you think that you’ll get a flu vaccine? A: From now on, yes. And one thing that's really influenced my values of vaccines, in college I had whooping cough and I was vaccinated against it as a child, but it was during 2004, so it was like during the outbreak and I went to Wisconsin, so a lot of people were having whooping cough and I got it and it was just horrible. (WFT-II).*

*Oh, I had-- Yeah, the only reason I started getting the flu shot is because I had it like two years in a row. And I was sick for like seven, eight days. I couldn’t eat. The only thing I could have was water. I was throwing up. I was doing everything. and I was just like, after this, I'm never, ever-- And, when you have children, if they get it, it passes through your house. So it just-- it’s just better if you-- when everybody gets it at the same time, that flu shot. And then the whole house is fine. Nobody had it this year. (AAFT-FG).*

*My thing is, I caught the flu years and years ago, real bad, you know. And, after the experience that I had of a week in the hospital and all like that, after that experience, yeah, give me the flu shot, you know. And then, you know, it’s just to me, preventative. (AAMT-FG).*

*Probably if I got the flu really badly one year then I would probably take it, the flu vaccine, after that. As far as I can remember I haven’t had a case of the flu, I don’t think (WMNT- II).*

*I know I haven’t in at least 15 years. I mean yes I have had the flu and I’ve had it pretty bad, and that in itself is a factor for me taking the vaccine is I don’t want that experience again (AAMT-II).*

Tradition of trusting the vaccine:

*But I come from a very medical background. My father’s a physician and I'm a nurse, so I'm very pro vaccine. And when I worked in a hospital, I was required to have it, especially with H1N1. They wouldn't let me enter unless I had it. (WFT-II)*

*I've literally probably gotten every vaccine that's ever been out there for, like, you know, infants, like DTaP and all of those. I don't know, I just think it’s something that's really important for a society to do in order to prevent, you know, terrible diseases that really caused a huge impact like a hundred years ago that we weren't able to do anything about. But today, we have the capability to really decrease these terrible diseases that killed so many people. So no, I'm not really religious at all. It’s just mainly preventing disease. (WFT-II)*

*Yeah, it is, it is. Because I feel like it is my duty if I can prevent other people from really getting sick, and myself from getting sick, that's really important. (WFT-II)*

*So, maybe that's why I'm so used to it. And growing up with my dad being a physician, it was always prioritized that we should get the vaccinations because he always said it was good and we shouldn’t-- he has his MPH, too, so it’s like very much-- so does my sister-- it’s very much like promoted in our family. (WFT-II)*

*See, I get all my other vaccines, too. I'm like dying to get the shingles one. She's like, “It's not approved for your age group.” And I'm like, “But I had the chicken pox. I know I'm going to get the shingles. I want it.” I wanted HPV, but I'm too old for that, I'm too young for shingles. I'm like, “I want it all,” you know? (WFT –II).*

**Original Codes and New Codes:** Original Codes recoded to fit the Three Cs Framework

	Original Codes
Complacency	Motivations: Passivity; Motivations: Unnecessary; “It’s my choice”; Anti-Doctor; Barriers: Convenience; Motivation: Flu susceptibility; Flu: Likelihood of contracting.
Convenience	Barriers: Convenience; Barriers: Financial; Culture: “too busy"; Motivation: External impetus; Motivation: Lack of barriers.
Confidence	“Guinea pigs”;“They” distrust; Conspiracy: Money; Government: Competence; Motivations: Unsure; Trust: Vaccine; Trust: Healthcare providers; Trust: Healthcare industry; Trust: government; Trust: Pharmaceutical companies; Naturalism: Beliefs; Race: Tuskegee; Motivations: Fixed Beliefs; Motivations: Pro-vaccine; Motivation: Initiative; Motivations: Risk Comparison; Vaccine: Side Effects; Vaccine: safety.
